# *N*-(2,3-Dichloro­phen­yl)-4-methyl­benzene­sulfonamide

**DOI:** 10.1107/S1600536810044375

**Published:** 2010-11-06

**Authors:** K. Shakuntala, Sabine Foro, B. Thimme Gowda, P. G. Nirmala, Hartmut Fuess

**Affiliations:** a Department of Chemistry, Mangalore University, Mangalagangotri 574 199, Mangalore, India; b Institute of Materials Science, Darmstadt University of Technology, Petersenstrasse 23, D-64287 Darmstadt, Germany

## Abstract

The title compound, C_13_H_11_Cl_2_NO_2_S, contains two molecules in the asymmetric unit in which the dihedral angles between the benzene rings are 76.0 (1) and 79.9 (1)°. The conformations of the N—H bonds with respect to their adjacent ortho-chlorine atoms are syn. In the crystal, N—H⋯O hydrogen bonds link the molecules into dimers.

## Related literature

For our study of the effect of substituents on the structures of *N*-(ar­yl)aryl­sulfonamides, see: Gowda *et al.* (2009 ▶, 2010*a* ▶,*b* ▶). For related structures, see: Gelbrich *et al.* (2007 ▶); Perlovich *et al.* (2006 ▶).
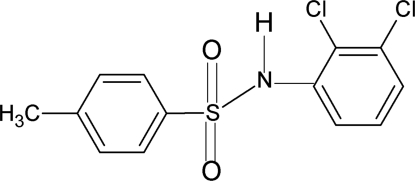


## Experimental

### 

#### Crystal data

C_13_H_11_Cl_2_NO_2_S
*M* *_r_* = 316.19
Monoclinic, 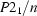

*a* = 14.035 (3) Å
*b* = 12.386 (2) Å
*c* = 15.993 (3) Å
β = 98.30 (1)°
*V* = 2751.1 (9) Å^3^
*Z* = 8
Cu *K*α radiation
μ = 5.64 mm^−1^
*T* = 299 K
0.50 × 0.45 × 0.40 mm

#### Data collection

Enraf–Nonius CAD-4 diffractometer
9784 measured reflections
4903 independent reflections
4359 reflections with *I* > 2σ(*I*)
*R*_int_ = 0.131
3 standard reflections every 120 min  intensity decay: 1.0%

#### Refinement

*R*[*F* ^2^ > 2σ(*F* ^2^)] = 0.065
*wR*(*F* ^2^) = 0.176
*S* = 1.11
4903 reflections
352 parameters
2 restraints
H atoms treated by a mixture of independent and constrained refinement
Δρ_max_ = 0.81 e Å^−3^
Δρ_min_ = −0.56 e Å^−3^

### 

Data collection: *CAD-4-PC* (Enraf–Nonius, 1996 ▶); cell refinement: *CAD-4-PC*; data reduction: *REDU4* (Stoe & Cie, 1987 ▶); program(s) used to solve structure: *SHELXS97* (Sheldrick, 2008 ▶); program(s) used to refine structure: *SHELXL97* (Sheldrick, 2008 ▶); molecular graphics: *PLATON* (Spek, 2009 ▶); software used to prepare material for publication: *SHELXL97*.

## Supplementary Material

Crystal structure: contains datablocks I, global. DOI: 10.1107/S1600536810044375/bq2248sup1.cif

Structure factors: contains datablocks I. DOI: 10.1107/S1600536810044375/bq2248Isup2.hkl

Additional supplementary materials:  crystallographic information; 3D view; checkCIF report

## Figures and Tables

**Table 1:** Hydrogen-bond geometry (Å, °)

*D*—H⋯*A*	*D*—H	H⋯*A*	*D*⋯*A*	*D*—H⋯*A*
N1—H1*N*⋯O3	0.84 (2)	2.29 (2)	3.086 (3)	160 (3)
N2—H2*N*⋯O2	0.85 (2)	2.32 (2)	3.104 (3)	154 (3)

**Fig. 1.**
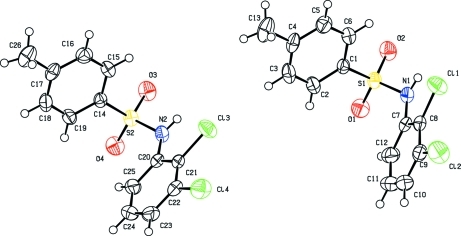
Molecular structure of (I), showing the atom labeling scheme and displacement ellipsoids are drawn at the 50% probability level.

**Fig. 2.**
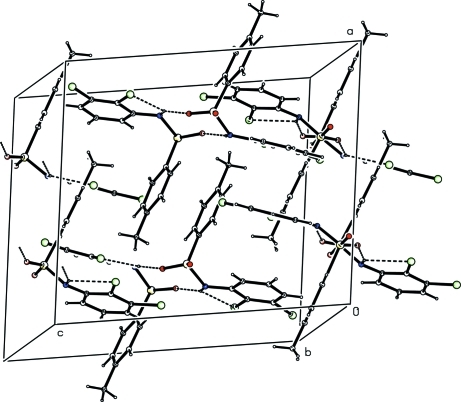
Molecular packing of (I) with hydrogen bonding shown as dashed lines.

## supplementary crystallographic information

### Comment

As part of a study of the substituent effects on the crystal structures of *N*-(aryl)-arylsulfonamides (Gowda *et al.*, 2009; 2010**a*, b*), in the present work, the structure of 4-methyl-*N*-(2,3-dichlorophenyl)- benzenesulfonamide (I) has been determined. The asymmetric unit of (I) contains two independent molecules. In one of the molecules, conformation of the N—C bond in the C—SO_2_—NH—C segment of the structure has *gauche* torsions with respect to the S═O bonds (Fig. 1). The molecules are bent at the S atoms with the C—SO_2_—NH—C torsion angles of 65.4 (2) and -61.7 (2). The conformations of the N—H bonds and the *ortho*-chloro groups in the anilino benzene rings are *syn* to each other. The benzene rings in the title compound are tilted relative to each other by 76.0 (1)° (molecule 1) and 79.9 (1)° (molecule 2). The other bond parameters in (I) are similar to those observed in 4-methyl-*N*-(2-chlorophenyl)- benzenesulfonamide (Gowda *et al.*, 2010*a*), 4-methyl-*N*-(3-chlorophenyl)benzenesulfonamide (Gowda *et al.*, 2010*b*), 4-methyl-*N*-(3,5-dichlorophenyl)benzenesulfonamide (Gowda *et al.*, 2009) and other aryl sulfonamides (Perlovich *et al.*, 2006; Gelbrich *et al.*, 2007). The N—H···O hydrogen bonds (Table 1) pack the molecules into infinite chains in the direction of *a*- axis (Fig. 2).

### Experimental

The solution of toluene (10 ml) in chloroform (40 ml) was treated dropwise with chlorosulfonic acid (25 ml) at 0 ° C. After the initial evolution of hydrogen chloride subsided, the reaction mixture was brought to room temperature and poured into crushed ice in a beaker. The chloroform layer was separated, washed with cold water and allowed to evaporate slowly. The residual 4-methylbenzenesulfonylchloride was treated with 2,3-dichloroaniline in the stoichiometric ratio and boiled for ten minutes. The reaction mixture was then cooled to room temperature and added to ice cold water (100 ml). The resultant 4-methyl-*N*-(2,3-dichlorophenyl)benzenesulfonamide was filtered under suction and washed thoroughly with cold water. It was then recrystallized to constant melting point from dilute ethanol. The purity of the compound was checked and characterized by recording its infrared and NMR spectra. The single crystals used in X-ray diffraction studies were grown in ethanolic solution by slow evaporation at room temperature.

### Refinement

The H atoms of the NH groups were located in a difference map and later restrained to N—H = 0.86 (2) %A. The other H atoms were positioned with idealized geometry using a riding model with C—H = 0.93–0.96 Å. All H atoms were refined with isotropic displacement parameters (set to 1.2 times of the *U*_eq_ of the parent atom).

### Crystal data

**Table:**

C_13_H_11_Cl_2_NO_2_S	*F*(000) = 1296
*M**_r_* = 316.19	*D*_x_ = 1.527 Mg m^−^^3^
Monoclinic, *P*2_1_/*n*	Cu *K*α radiation, λ = 1.54180 Å
Hall symbol: -P 2yn	Cell parameters from 25 reflections
*a* = 14.035 (3) Å	θ = 5.6–19.2°
*b* = 12.386 (2) Å	µ = 5.64 mm^−^^1^
*c* = 15.993 (3) Å	*T* = 299 K
β = 98.30 (1)°	Prism, colourless
*V* = 2751.1 (9) Å^3^	0.50 × 0.45 × 0.40 mm
*Z* = 8	

### Data collection

**Table:**

Enraf–Nonius CAD-4 diffractometer	*R*_int_ = 0.131
Radiation source: fine-focus sealed tube	θ_max_ = 67.0°, θ_min_ = 3.9°
graphite	*h* = −16→16
ω/2θ scans	*k* = −14→0
9784 measured reflections	*l* = −19→19
4903 independent reflections	3 standard reflections every 120 min
4359 reflections with *I* > 2σ(*I*)	intensity decay: 1.0%

### Refinement

**Table:**

Refinement on *F*^2^	Secondary atom site location: difference Fourier map
Least-squares matrix: full	Hydrogen site location: inferred from neighbouring sites
*R*[*F*^2^ > 2σ(*F*^2^)] = 0.065	H atoms treated by a mixture of independent and constrained refinement
*wR*(*F*^2^) = 0.176	*w* = 1/[σ^2^(*F*_o_^2^) + (0.0914*P*)^2^ + 0.5192*P*] where *P* = (*F*_o_^2^ + 2*F*_c_^2^)/3
*S* = 1.11	(Δ/σ)_max_ < 0.001
4903 reflections	Δρ_max_ = 0.81 e Å^−^^3^
352 parameters	Δρ_min_ = −0.56 e Å^−^^3^
2 restraints	Extinction correction: *SHELXL97* (Sheldrick, 2008), Fc^*^=kFc[1+0.001xFc^2^λ^3^/sin(2θ)]^-1/4^
Primary atom site location: structure-invariant direct methods	Extinction coefficient: 0.0038 (4)

### Special details

**Table:**

Geometry. All e.s.d.'s (except the e.s.d. in the dihedral angle between two l.s. planes) are estimated using the full covariance matrix. The cell e.s.d.'s are taken into account individually in the estimation of e.s.d.'s in distances, angles and torsion angles; correlations between e.s.d.'s in cell parameters are only used when they are defined by crystal symmetry. An approximate (isotropic) treatment of cell e.s.d.'s is used for estimating e.s.d.'s involving l.s. planes.
Refinement. Refinement of *F*^2^ against ALL reflections. The weighted *R*-factor *wR* and goodness of fit *S* are based on *F*^2^, conventional *R*-factors *R* are based on *F*, with *F* set to zero for negative *F*^2^. The threshold expression of *F*^2^ > σ(*F*^2^) is used only for calculating *R*-factors(gt) *etc*. and is not relevant to the choice of reflections for refinement. *R*-factors based on *F*^2^ are statistically about twice as large as those based on *F*, and *R*- factors based on ALL data will be even larger.

### Fractional atomic coordinates and isotropic or equivalent isotropic displacement parameters (Å^2^)

**Table:**

	*x*	*y*	*z*	*U*_iso_*/*U*_eq_	
C1	0.88559 (18)	0.0982 (2)	0.42938 (14)	0.0312 (6)	
C2	0.9254 (2)	0.0053 (2)	0.40037 (17)	0.0404 (6)	
H2	0.8955	−0.0612	0.4038	0.048*	
C3	1.0094 (2)	0.0132 (3)	0.36653 (18)	0.0473 (7)	
H3	1.0361	−0.0489	0.3468	0.057*	
C4	1.05552 (19)	0.1107 (3)	0.36089 (16)	0.0412 (7)	
C5	1.0132 (2)	0.2032 (3)	0.38929 (19)	0.0453 (7)	
H5	1.0423	0.2700	0.3849	0.054*	
C6	0.9292 (2)	0.1972 (2)	0.42354 (18)	0.0413 (6)	
H6	0.9020	0.2592	0.4427	0.050*	
C7	0.66060 (17)	0.0946 (2)	0.32818 (17)	0.0353 (6)	
C8	0.64433 (18)	0.1597 (2)	0.25643 (17)	0.0375 (6)	
C9	0.6067 (2)	0.1151 (3)	0.17875 (18)	0.0428 (7)	
C10	0.5881 (2)	0.0062 (3)	0.1720 (2)	0.0578 (9)	
H10	0.5641	−0.0237	0.1199	0.069*	
C11	0.6048 (3)	−0.0574 (3)	0.2417 (3)	0.0637 (10)	
H11	0.5925	−0.1311	0.2365	0.076*	
C12	0.6398 (2)	−0.0151 (3)	0.3207 (2)	0.0530 (8)	
H12	0.6493	−0.0597	0.3680	0.064*	
C13	1.1486 (2)	0.1179 (4)	0.3250 (2)	0.0625 (10)	
H13A	1.1354	0.1367	0.2662	0.075*	
H13B	1.1809	0.0495	0.3311	0.075*	
H13C	1.1888	0.1723	0.3548	0.075*	
N1	0.69333 (16)	0.1410 (2)	0.40784 (15)	0.0379 (5)	
H1N	0.700 (3)	0.2081 (15)	0.410 (2)	0.045*	
O1	0.75897 (16)	−0.02137 (18)	0.48773 (13)	0.0491 (5)	
O2	0.78830 (16)	0.16207 (18)	0.54657 (12)	0.0470 (5)	
Cl1	0.67147 (7)	0.29473 (6)	0.26491 (5)	0.0542 (3)	
Cl2	0.58337 (8)	0.19562 (9)	0.09061 (5)	0.0690 (3)	
S1	0.77998 (4)	0.08962 (6)	0.47581 (4)	0.0344 (2)	
C14	0.62488 (18)	0.4300 (2)	0.57233 (14)	0.0302 (5)	
C15	0.5861 (2)	0.3271 (2)	0.57427 (16)	0.0372 (6)	
H15	0.6173	0.2680	0.5548	0.045*	
C16	0.5007 (2)	0.3142 (2)	0.60547 (18)	0.0420 (6)	
H16	0.4740	0.2455	0.6066	0.050*	
C17	0.45287 (19)	0.4019 (3)	0.63566 (16)	0.0397 (6)	
C18	0.4933 (2)	0.5035 (3)	0.63213 (18)	0.0437 (7)	
H18	0.4619	0.5629	0.6510	0.052*	
C19	0.5792 (2)	0.5186 (2)	0.60121 (17)	0.0383 (6)	
H19	0.6059	0.5872	0.5998	0.046*	
C20	0.83949 (17)	0.4415 (2)	0.68958 (16)	0.0322 (5)	
C21	0.87507 (17)	0.3732 (2)	0.75535 (16)	0.0318 (5)	
C22	0.89966 (18)	0.4133 (2)	0.83721 (17)	0.0375 (6)	
C23	0.8875 (2)	0.5210 (3)	0.8543 (2)	0.0484 (7)	
H23	0.9037	0.5475	0.9089	0.058*	
C24	0.8515 (2)	0.5880 (3)	0.7900 (2)	0.0509 (7)	
H24	0.8421	0.6605	0.8014	0.061*	
C25	0.8284 (2)	0.5505 (2)	0.70714 (19)	0.0440 (7)	
H25	0.8056	0.5982	0.6639	0.053*	
C26	0.3602 (2)	0.3853 (3)	0.6705 (2)	0.0560 (9)	
H26A	0.3739	0.3697	0.7298	0.067*	
H26B	0.3257	0.3259	0.6418	0.067*	
H26C	0.3218	0.4495	0.6620	0.067*	
N2	0.81882 (17)	0.39956 (19)	0.60644 (14)	0.0370 (5)	
H2N	0.826 (2)	0.3316 (16)	0.603 (2)	0.044*	
O3	0.73700 (16)	0.37678 (18)	0.46284 (11)	0.0475 (5)	
O4	0.75082 (16)	0.55963 (18)	0.52425 (13)	0.0480 (5)	
Cl3	0.88652 (6)	0.23746 (6)	0.73614 (5)	0.0526 (3)	
Cl4	0.94255 (7)	0.32756 (8)	0.91866 (5)	0.0600 (3)	
S2	0.73465 (5)	0.44706 (5)	0.53342 (4)	0.0342 (2)	

### Atomic displacement parameters (Å^2^)

**Table:**

	*U*^11^	*U*^22^	*U*^33^	*U*^12^	*U*^13^	*U*^23^
C1	0.0288 (12)	0.0371 (14)	0.0268 (11)	0.0076 (10)	0.0013 (10)	−0.0017 (10)
C2	0.0439 (15)	0.0369 (14)	0.0404 (13)	0.0106 (12)	0.0061 (12)	−0.0029 (11)
C3	0.0477 (16)	0.0519 (17)	0.0430 (14)	0.0209 (15)	0.0094 (13)	−0.0032 (13)
C4	0.0297 (13)	0.0630 (19)	0.0302 (12)	0.0149 (13)	0.0021 (10)	0.0059 (12)
C5	0.0375 (15)	0.0479 (16)	0.0507 (16)	0.0007 (13)	0.0070 (13)	0.0035 (13)
C6	0.0379 (14)	0.0394 (15)	0.0475 (14)	0.0070 (12)	0.0089 (12)	−0.0044 (12)
C7	0.0203 (11)	0.0388 (14)	0.0458 (14)	0.0026 (10)	0.0010 (10)	−0.0021 (11)
C8	0.0250 (11)	0.0403 (14)	0.0472 (14)	0.0050 (11)	0.0055 (11)	−0.0079 (12)
C9	0.0314 (13)	0.0523 (17)	0.0436 (14)	0.0073 (13)	0.0021 (11)	−0.0059 (13)
C10	0.0478 (17)	0.056 (2)	0.0638 (19)	0.0036 (16)	−0.0109 (16)	−0.0218 (17)
C11	0.060 (2)	0.0422 (17)	0.080 (2)	−0.0048 (16)	−0.0183 (19)	−0.0116 (17)
C12	0.0467 (17)	0.0407 (16)	0.067 (2)	−0.0051 (14)	−0.0069 (16)	0.0032 (15)
C13	0.0407 (17)	0.099 (3)	0.0501 (17)	0.0165 (18)	0.0151 (14)	0.0137 (19)
N1	0.0304 (11)	0.0368 (12)	0.0465 (12)	0.0050 (10)	0.0062 (10)	−0.0011 (10)
O1	0.0523 (12)	0.0440 (12)	0.0517 (11)	−0.0005 (10)	0.0096 (10)	0.0120 (9)
O2	0.0531 (12)	0.0547 (12)	0.0342 (9)	0.0095 (10)	0.0102 (9)	−0.0046 (9)
Cl1	0.0739 (6)	0.0381 (4)	0.0496 (4)	0.0011 (3)	0.0049 (4)	0.0001 (3)
Cl2	0.0888 (7)	0.0725 (6)	0.0424 (4)	0.0130 (5)	−0.0019 (4)	0.0002 (4)
S1	0.0341 (4)	0.0386 (4)	0.0314 (3)	0.0055 (3)	0.0076 (3)	0.0024 (2)
C14	0.0315 (12)	0.0331 (12)	0.0247 (10)	0.0062 (10)	−0.0002 (10)	0.0018 (9)
C15	0.0435 (14)	0.0300 (12)	0.0386 (12)	0.0062 (12)	0.0076 (12)	−0.0001 (11)
C16	0.0411 (15)	0.0397 (15)	0.0445 (14)	−0.0033 (13)	0.0034 (12)	0.0034 (12)
C17	0.0295 (13)	0.0564 (17)	0.0308 (12)	0.0052 (12)	−0.0035 (10)	0.0041 (12)
C18	0.0374 (14)	0.0464 (16)	0.0472 (15)	0.0149 (13)	0.0062 (12)	−0.0023 (13)
C19	0.0418 (14)	0.0304 (13)	0.0410 (13)	0.0066 (12)	0.0005 (12)	−0.0027 (11)
C20	0.0214 (11)	0.0346 (13)	0.0403 (13)	−0.0015 (10)	0.0036 (10)	−0.0017 (11)
C21	0.0220 (11)	0.0311 (12)	0.0424 (13)	0.0003 (10)	0.0047 (10)	−0.0023 (11)
C22	0.0259 (11)	0.0449 (15)	0.0406 (13)	−0.0018 (11)	0.0007 (10)	0.0028 (12)
C23	0.0416 (15)	0.0538 (18)	0.0476 (15)	−0.0032 (14)	−0.0011 (13)	−0.0150 (14)
C24	0.0511 (17)	0.0367 (15)	0.0613 (18)	0.0000 (14)	−0.0044 (15)	−0.0127 (14)
C25	0.0441 (15)	0.0334 (14)	0.0509 (15)	0.0025 (12)	−0.0055 (13)	0.0003 (12)
C26	0.0375 (15)	0.083 (3)	0.0479 (16)	0.0029 (16)	0.0082 (13)	0.0082 (17)
N2	0.0370 (12)	0.0364 (12)	0.0379 (11)	0.0087 (10)	0.0061 (10)	−0.0006 (9)
O3	0.0581 (13)	0.0548 (12)	0.0315 (9)	0.0080 (11)	0.0128 (9)	−0.0008 (9)
O4	0.0569 (12)	0.0404 (11)	0.0480 (11)	−0.0015 (10)	0.0121 (10)	0.0119 (9)
Cl3	0.0709 (5)	0.0328 (4)	0.0515 (4)	0.0090 (3)	0.0001 (4)	0.0004 (3)
Cl4	0.0666 (5)	0.0630 (5)	0.0455 (4)	0.0009 (4)	−0.0081 (4)	0.0085 (4)
S2	0.0375 (4)	0.0354 (4)	0.0306 (3)	0.0034 (3)	0.0077 (3)	0.0043 (2)

### Geometric parameters (Å, °)

**Table:**

C1—C6	1.380 (4)	C14—C19	1.384 (4)
C1—C2	1.387 (4)	C14—C15	1.388 (4)
C1—S1	1.754 (2)	C14—S2	1.756 (2)
C2—C3	1.370 (4)	C15—C16	1.373 (4)
C2—H2	0.9300	C15—H15	0.9300
C3—C4	1.380 (5)	C16—C17	1.399 (4)
C3—H3	0.9300	C16—H16	0.9300
C4—C5	1.396 (4)	C17—C18	1.385 (4)
C4—C13	1.503 (4)	C17—C26	1.500 (4)
C5—C6	1.371 (4)	C18—C19	1.379 (4)
C5—H5	0.9300	C18—H18	0.9300
C6—H6	0.9300	C19—H19	0.9300
C7—C12	1.392 (4)	C20—C21	1.386 (4)
C7—C8	1.394 (4)	C20—C25	1.392 (4)
C7—N1	1.413 (4)	C20—N2	1.418 (3)
C8—C9	1.392 (4)	C21—C22	1.396 (4)
C8—Cl1	1.716 (3)	C21—Cl3	1.721 (3)
C9—C10	1.375 (5)	C22—C23	1.378 (4)
C9—Cl2	1.719 (3)	C22—Cl4	1.720 (3)
C10—C11	1.358 (6)	C23—C24	1.361 (5)
C10—H10	0.9300	C23—H23	0.9300
C11—C12	1.390 (5)	C24—C25	1.397 (4)
C11—H11	0.9300	C24—H24	0.9300
C12—H12	0.9300	C25—H25	0.9300
C13—H13A	0.9600	C26—H26A	0.9600
C13—H13B	0.9600	C26—H26B	0.9600
C13—H13C	0.9600	C26—H26C	0.9600
N1—S1	1.638 (2)	N2—S2	1.645 (2)
N1—H1N	0.837 (18)	N2—H2N	0.851 (18)
O1—S1	1.425 (2)	O3—S2	1.430 (2)
O2—S1	1.436 (2)	O4—S2	1.424 (2)
			
C6—C1—C2	120.7 (2)	C19—C14—C15	121.2 (2)
C6—C1—S1	119.5 (2)	C19—C14—S2	119.7 (2)
C2—C1—S1	119.8 (2)	C15—C14—S2	119.1 (2)
C3—C2—C1	118.9 (3)	C16—C15—C14	118.8 (2)
C3—C2—H2	120.5	C16—C15—H15	120.6
C1—C2—H2	120.5	C14—C15—H15	120.6
C2—C3—C4	121.8 (3)	C15—C16—C17	121.5 (3)
C2—C3—H3	119.1	C15—C16—H16	119.3
C4—C3—H3	119.1	C17—C16—H16	119.3
C3—C4—C5	118.1 (3)	C18—C17—C16	118.1 (3)
C3—C4—C13	121.2 (3)	C18—C17—C26	121.5 (3)
C5—C4—C13	120.6 (3)	C16—C17—C26	120.4 (3)
C6—C5—C4	121.1 (3)	C19—C18—C17	121.5 (3)
C6—C5—H5	119.5	C19—C18—H18	119.3
C4—C5—H5	119.5	C17—C18—H18	119.3
C5—C6—C1	119.4 (3)	C18—C19—C14	118.9 (3)
C5—C6—H6	120.3	C18—C19—H19	120.6
C1—C6—H6	120.3	C14—C19—H19	120.6
C12—C7—C8	119.1 (3)	C21—C20—C25	118.5 (2)
C12—C7—N1	120.8 (3)	C21—C20—N2	119.3 (2)
C8—C7—N1	120.0 (3)	C25—C20—N2	122.1 (2)
C9—C8—C7	120.0 (3)	C20—C21—C22	120.5 (2)
C9—C8—Cl1	120.6 (2)	C20—C21—Cl3	119.61 (19)
C7—C8—Cl1	119.4 (2)	C22—C21—Cl3	119.9 (2)
C10—C9—C8	120.2 (3)	C23—C22—C21	120.6 (3)
C10—C9—Cl2	119.5 (2)	C23—C22—Cl4	119.1 (2)
C8—C9—Cl2	120.3 (2)	C21—C22—Cl4	120.2 (2)
C11—C10—C9	119.8 (3)	C24—C23—C22	118.9 (3)
C11—C10—H10	120.1	C24—C23—H23	120.5
C9—C10—H10	120.1	C22—C23—H23	120.5
C10—C11—C12	121.6 (3)	C23—C24—C25	121.7 (3)
C10—C11—H11	119.2	C23—C24—H24	119.2
C12—C11—H11	119.2	C25—C24—H24	119.2
C11—C12—C7	119.2 (3)	C20—C25—C24	119.7 (3)
C11—C12—H12	120.4	C20—C25—H25	120.1
C7—C12—H12	120.4	C24—C25—H25	120.1
C4—C13—H13A	109.5	C17—C26—H26A	109.5
C4—C13—H13B	109.5	C17—C26—H26B	109.5
H13A—C13—H13B	109.5	H26A—C26—H26B	109.5
C4—C13—H13C	109.5	C17—C26—H26C	109.5
H13A—C13—H13C	109.5	H26A—C26—H26C	109.5
H13B—C13—H13C	109.5	H26B—C26—H26C	109.5
C7—N1—S1	123.66 (19)	C20—N2—S2	124.27 (18)
C7—N1—H1N	117 (2)	C20—N2—H2N	114 (2)
S1—N1—H1N	107 (2)	S2—N2—H2N	113 (2)
O1—S1—O2	119.51 (13)	O4—S2—O3	119.53 (12)
O1—S1—N1	108.38 (13)	O4—S2—N2	108.34 (13)
O2—S1—N1	104.38 (12)	O3—S2—N2	104.28 (12)
O1—S1—C1	108.59 (13)	O4—S2—C14	108.40 (13)
O2—S1—C1	108.57 (12)	O3—S2—C14	109.14 (13)
N1—S1—C1	106.70 (12)	N2—S2—C14	106.39 (11)
			
C6—C1—C2—C3	0.7 (4)	C19—C14—C15—C16	0.1 (4)
S1—C1—C2—C3	−178.1 (2)	S2—C14—C15—C16	179.4 (2)
C1—C2—C3—C4	0.3 (4)	C14—C15—C16—C17	−0.5 (4)
C2—C3—C4—C5	−1.3 (4)	C15—C16—C17—C18	1.0 (4)
C2—C3—C4—C13	178.9 (3)	C15—C16—C17—C26	−179.2 (3)
C3—C4—C5—C6	1.4 (4)	C16—C17—C18—C19	−1.1 (4)
C13—C4—C5—C6	−178.8 (3)	C26—C17—C18—C19	179.1 (3)
C4—C5—C6—C1	−0.5 (4)	C17—C18—C19—C14	0.7 (4)
C2—C1—C6—C5	−0.5 (4)	C15—C14—C19—C18	−0.2 (4)
S1—C1—C6—C5	178.3 (2)	S2—C14—C19—C18	−179.5 (2)
C12—C7—C8—C9	0.8 (4)	C25—C20—C21—C22	−0.4 (4)
N1—C7—C8—C9	−176.2 (2)	N2—C20—C21—C22	177.4 (2)
C12—C7—C8—Cl1	−179.0 (2)	C25—C20—C21—Cl3	178.2 (2)
N1—C7—C8—Cl1	4.0 (3)	N2—C20—C21—Cl3	−4.0 (3)
C7—C8—C9—C10	−1.9 (4)	C20—C21—C22—C23	1.1 (4)
Cl1—C8—C9—C10	177.9 (2)	Cl3—C21—C22—C23	−177.6 (2)
C7—C8—C9—Cl2	178.22 (19)	C20—C21—C22—Cl4	179.30 (18)
Cl1—C8—C9—Cl2	−2.0 (3)	Cl3—C21—C22—Cl4	0.7 (3)
C8—C9—C10—C11	1.2 (5)	C21—C22—C23—C24	−0.3 (4)
Cl2—C9—C10—C11	−178.9 (3)	Cl4—C22—C23—C24	−178.5 (2)
C9—C10—C11—C12	0.5 (6)	C22—C23—C24—C25	−1.2 (5)
C10—C11—C12—C7	−1.7 (5)	C21—C20—C25—C24	−1.0 (4)
C8—C7—C12—C11	1.0 (4)	N2—C20—C25—C24	−178.8 (3)
N1—C7—C12—C11	177.9 (3)	C23—C24—C25—C20	1.9 (5)
C12—C7—N1—S1	47.9 (3)	C21—C20—N2—S2	150.1 (2)
C8—C7—N1—S1	−135.2 (2)	C25—C20—N2—S2	−32.2 (3)
C7—N1—S1—O1	−51.4 (2)	C20—N2—S2—O4	54.7 (2)
C7—N1—S1—O2	−179.8 (2)	C20—N2—S2—O3	−177.0 (2)
C7—N1—S1—C1	65.4 (2)	C20—N2—S2—C14	−61.7 (2)
C6—C1—S1—O1	−169.9 (2)	C19—C14—S2—O4	−10.5 (2)
C2—C1—S1—O1	8.9 (3)	C15—C14—S2—O4	170.1 (2)
C6—C1—S1—O2	−38.5 (3)	C19—C14—S2—O3	−142.2 (2)
C2—C1—S1—O2	140.3 (2)	C15—C14—S2—O3	38.4 (2)
C6—C1—S1—N1	73.5 (2)	C19—C14—S2—N2	105.8 (2)
C2—C1—S1—N1	−107.8 (2)	C15—C14—S2—N2	−73.6 (2)

### Hydrogen-bond geometry (Å, °)

**Table:**

*D*—H···*A*	*D*—H	H···*A*	*D*···*A*	*D*—H···*A*
N1—H1N···O3	0.84 (2)	2.29 (2)	3.086 (3)	160 (3)
N1—H1N···Cl1	0.84 (2)	2.54 (3)	2.957 (2)	112 (3)
N2—H2N···O2	0.85 (2)	2.32 (2)	3.104 (3)	154 (3)
N2—H2N···Cl3	0.85 (2)	2.46 (3)	2.944 (2)	117 (3)
